# Editorial: Generation of Functional tissues from human pluripotent stem cells

**DOI:** 10.3389/fcell.2023.1155560

**Published:** 2023-02-08

**Authors:** Dongsheng Guo, Xianming Wang, Ke Huang

**Affiliations:** ^1^ Wellstone Muscular Dystrophy Program, Department of Neurology, University of Massachusetts Chan Medical School, Worcester, MA, United States; ^2^ Developmental Biology Program, Memorial Sloan Kettering Cancer Center, New York, NY, United States; ^3^ The Seventh Affiliated Hospital, Sun Yat-sen University, Shenzhen, Guangdong, China

**Keywords:** human pluripotent stem cells (hPSCs), human embryonic stem cells (hESCs), human induced pluripotent stem cells, differentiation, functional tissue cells

Dear readers, Human pluripotent stem cells (hPSCs), including embryonic stem cells isolated from the inner cell mass of a blastocyst (Thomson et al., 1998) and induced pluripotent stem cells (iPSCs) reprogrammed from somatic cells (Takahashi et al., 2007), could self-renew *in vitro* and have the potential to differentiate into any cell types of three germ layers. hPSCs and their derived cells could be used for genetic disease modeling, pathogenic mechanism investigation, and drug screening, and also provide unlimited cell resources for regenerative medicine. However, due to the differences between *in vitro* stem cell differentiation and *in vivo* development, most hPSCs-derived cells resemble fetal-stage cells and are not fully mature. Worldwide efforts are still on the way to generate functional tissue cells from stem cells. For this aim, we launched the specific Research Topic “Generation of Functional Tissues from Human Pluripotent Stem Cells” to highlight the latest progress to make functional cells from hPSCs and their applications. Throughout this Research Topic, we compiled various research papers from expert laboratories around the world within this exciting field of research.

## New strategy to promote hPSCs differentiation into beta cells

Diabetes research and cell treatment can benefit from the differentiation of hPSCs into beta cells in dish. Using static microwells instead of rotating suspensions, Fanuzzi et al. reported generating beta cells from human induced pluripotent stem cells (iPSCs) with higher success rates. This report provides an efficient platform for creating size-controlled aggregates that are reproducible and standardized.

## New models for investigating cell-cell interaction *in vitro*

Physiological and pathological states are influenced by cellular interactions between different cells (Armingol et al., 2021). hPSCs and specific differentiations are useful tools for capturing cell-cell interactions and investigating biological mechanisms. Using hiPSCs differentiated neural progenitor cells (NPCs), Lilienberg et al. examined the modulatory effects of microglia on polarization and differentiation, demonstrating the complexity of the effect of microglia on neurite generation and differentiation, as well as providing a better understanding of this basic cell biological phenomenon. Massih et al. used iPSC-derived motoneurons (MNs) and 3D muscle tissue derived from myoblasts in a 3D neuromuscular cell culture system to study human physiology *in vitro*.

## New methods for functional assay of hPSCs derived cells

The patch-clamp method is widely used to record action potentials to assess the electrical function of cardiomyocytes. However, this method is very labor-intensive and not suitable for high-throughput monitoring. Zhang et al. developed a powerful platform for high-throughput and repeatable action potential measurements in hiPSC-derived ventricular, atrial, and nodal cardiomyocytes in a 2D monolayer and single cardiomyocytes in a 3D context by inserting genetically encoded voltage indicator in the safe harbor locus of hiPSCs. This platform could facilitate the development and evaluation of more functionally mature, biologically relevant engineered cardiac tissues.

hPSCs-derived kidney organoids have opened a new world of possibilities for developing preclinical kidney models and immunocompatible kidney tissues for regeneration. Although organoids resemble native nephrons, which consist of tubules and filtration units, little is known about their function. Rizki-Safitri et al. established a live functional assessment in 3D kidney organoids, making it possible to study how organoids function in health and diseases.

Thanks to the authors for their outstanding contributions and the reviewers for their constructive comments, we were able to put together such an excellent Research Topic of works in this field.

## Perspectives

Though remarkable progress has been made, we are still facing big challenges in making functional cells for most cell types due to the lack of understanding of how these cells develop *in vivo*. Advances in various sequencing technologies and multi-omics analysis offer unprecedented insight into the developmental process of different types of cells. These understanding will eventually guide the differentiation of hPSCs into functional cells.

In light of the FDA’s recent decision not to require all drugs to be tested on animals before human trials (Wadman, 2023), hPSCs derived-function cells or organoids (3D) are good alternatives for drugs and therapeutic tests. By mimicking the microenvironment *in vivo*, new biomaterials will promote hPSCs differentiation dramatically. Using stem cells, scientists can generate fully functional tissues in the future.
